# An Evaluation of Immature Granulocytes as Predictors of Malignancy in Patients with Atypia of Undetermined Significance Thyroid Nodules

**DOI:** 10.1055/a-2827-6793

**Published:** 2026-03-16

**Authors:** Damla Tüfekçi, Hasan Gucer

**Affiliations:** 1 175650 Endocrinology and Metabolism, Recep Tayyip Erdogan Universitesi, Rize, Turkey; 2 175650 Pathology, Recep Tayyip Erdogan Universitesi, Rize, Turkey

**Keywords:** atypia of undetermined significance, immature granulocyte, thyroid, malignancy

## Abstract

This retrospective study aimed to evaluate hematological and inflammatory markers as predictors of thyroid cancer in patients with atypia of undetermined significance thyroid nodules. A total of 174 patients with atypia of undetermined significance who underwent thyroidectomy were included. Pre- and postoperative immature granulocyte counts, neutrophil-to-lymphocyte ratio, and platelet-to-lymphocyte ratio were analyzed after achieving euthyroid status. Propensity score matching for age and gender resulted in a final cohort of 128 patients (64 benign and 64 malignant). Static preoperative and postoperative immature granulocyte values did not differ significantly between the benign and malignant groups; however, the delta immature granulocyte value, defined as the change between pre- and postoperative measurements, was significantly lower in malignant cases ( *p* =0.007). Receiver operating characteristic analysis demonstrated an area under the curve of 0.651 at a cut-off value of≤− 0.01, with a sensitivity of 46.2% and a specificity of 79.2%. Univariate logistic regression revealed that delta immature granulocytes independently predicted malignancy in the overall cohort (odds ratio=3.273 and *p* =0.007) and in patients younger than 55 years (odds ratio=5.082 and *p* =0.007), whereas this association was not observed in patients aged 55 years and older. The neutrophil-to-lymphocyte and platelet-to-lymphocyte ratios were not significant predictors. These findings suggest that dynamic changes in immature granulocyte levels between the pre- and postoperative periods, rather than single-time-point measurements, may serve as a cost-effective and accessible complementary tool for malignancy prediction in atypia of undetermined significance thyroid nodules.

## Introduction

Thyroid tissue nodularity is a highly prevalent phenomenon in clinical practice, affecting up to 60% of adults in the general population when detected by ultrasound. 1​ Nodules are mostly benign (95–99%), but require evaluation to rule out malignancy (1–5% risk). 2​ Fine needle aspiration (FNA) biopsy remains the gold standard technique for the evaluation of thyroid nodules due to its high diagnostic accuracy of 86% sensitivity and 71% specificity. 3​

However, FNA has inherent limitations, particularly regarding inconclusive results; diagnoses of “atypia of undetermined significance” (AUS) or non-diagnostic nodules account for roughly 5–10% of all biopsies. 4​ These indeterminate results create significant uncertainty in clinical management and statistical evaluation. Although the American Thyroid Association guidelines recommend repeat FNA, diagnostic surgery, or molecular testing for these cases, 5​ ​6​ there remains an important gap: no molecular test can reliably and completely exclude malignancy, and such tests may be costly and available only in a limited number of centers in Turkey. Therefore, accessible and reliable adjunct markers are needed to help guide clinical decision-making in AUS nodules.

Cancer-related inflammation is known to be involved in carcinogenesis and the progression of neoplastic disease, including papillary thyroid carcinoma. 7​ ​8​ Immature granulocyte (IG) counts and percentages increase due to bone marrow activation in inflammatory states. 9​ We hypothesized that IG fractions (promyelocytes, myelocytes, and metamyelocytes) may reflect the inflammatory response associated with thyroid malignancy. The aim of this study was to evaluate the diagnostic utility of hematological indices, specifically IG fractions, as accessible predictive markers for malignancy in patients with thyroid nodules classified as AUS.

## Materials and methods

### Ethical considerations

This study was conducted in accordance with the Declaration of Helsinki and approved by the Rize Recep Tayyip Erdogan University Clinical Research Ethics Committee (Approval No: 2024/28; Date: 01.02.2024). Owing to the retrospective design, informed consent was waived. All data were anonymized, and the study complied with institutional and national regulations.

### Study design and patient selection

Three hundred thirteen patients diagnosed with AUS via FNA at the Rize Recep Tayyip Erdogan University Medical Pathology Department, between 2022 and 2025, were retrospectively selected and investigated in Turkey. Patients with additional diagnoses of cancer, rheumatological or inflammatory diseases, or severe organ failure, or with kidney transplantation, and pregnant women were excluded.

The FNA results were classified during the selection process according to the Bethesda System (version 2). One hundred seventy-four patients underwent surgery and had confirmed final histopathological examination results. These 174 patients were enrolled as the study participants.

### Hematological indices and laboratory data

IG values were determined using the closest preoperative laboratory data to the time of surgery and the values obtained when postoperative euthyroid status was achieved.

### Pathological and ultrasonographic data

The tumour size in patients with pathologically confirmed cancer after surgery was measured based on pathological data. The tumor size in patients with unconfirmed cancer was measured using preoperative ultrasonography. The primary tumour dimension was defined as the largest tumor size, consistent with standard practice in thyroid cancer screening. The classification of nodules by ultrasound was based on Europe (EU) thyroid imaging reporting and data system (TI-RADS).

For the definite analysis, patients with postoperative pathologically confirmed cancer were evaluated using preoperative imaging techniques (ultrasound or computed tomography) to confirm whether nodules reported as AUS prior to surgery could, in fact, have been identified as cancer. If nodules initially reported as AUS were not subsequently confirmed as tumours, these were not included in the study.

### Statistical analyses

Propensity score matching (PSM) analysis was first applied to yield balanced datasets by controlling for the confounding effects of age and gender between the benign and malignant groups. Matching scores were calculated using the logistic regression method. Matching was applied at a 1:1 ratio, with the nearest-neighbour and calliper values set to 0.2 for optimal matching.

Since both groups consisted of more than 50 patients, normality of data distribution was examined using the Kolmogorov–Smirnov test. The independent two-sample test was applied to compare normally distributed data between group pairs and the Mann–Whitney *U* test for non-normally distributed data. Categorical data were compared between the groups using Pearson’s chi-square test if all expected values were greater than 5 or Fisher’s exact test if any of the expected values were lower than 5. Multiple comparisons of ratios were performed using the Bonferroni-corrected *Z* test. Wilcoxon’s test was used to compare pre- and postoperative data that were not normally distributed. The effect of factors associated with malignancy was investigated using logistic regression analysis. Receiver operating characteristic (ROC) analysis was applied to investigate the diagnostic sufficiency of malignancy-related factors, and cut-off points were determined using the Youden index.

The analysis results were expressed as mean, standard deviation (mean±SD), and median and minimum and maximum (mean, min-max) values for quantitative data, and as frequency ( *n* ) and percentage (%) values for categorical data. *p* -Values of<0.05 were regarded as significant for all calculations. Statistical analyses were carried out using IBM SPSS version 26 and R software. Findings on R software were obtained via MatchIt and ggplot2 (packages).

## Results

A total of 174 patients were included in the study, with 109 (62.6%) patients in the benign group and 65 (37.3%) patients in the malignant group. After PSM to control for age and gender, 64 patients from each group were included in the final analysis, achieving balanced distributions between groups.

The mean age of 128 patients was 52.18 years, ranging from 13 to 79. The patients were 76.6% women and 23.4% men. Medication use distributions differed significantly between the patient groups ( *p* =0.012). No drugs were used by 67.2% of the benign group and 45.3% of the malignancy group. The proportion of patients using LT4 was 26.6% in the benign group and 51.6% in the malignancy group. However, no significant differences were observed in terms of demographic characteristics or preoperative clinical findings between the patient groups ( *p* >0.05; Table 1 ).

**Table:** **Table 1** Demographic characteristics and preoperative clinical and laboratory findings of the study population

	Benign ( *n* =64)	Malignant ( *n* =64)	Total ( *N* =128)	*p*
Sex (%)				
Female	49 (76.6)	49 (76.6)	98 (76.6)	1.000 ^x^
Male	15 (23.4)	15 (23.4)	30 (23.4)	
Age (y)	52.42±10.41	51.94±10.12	52.18±10.23	0.790 ^t^
Medication use				
None	43 (67.2) ^a^	29 (45.3) ^b^	72 (56.3)	**0.012** ^f^
Levothyroxine	17 (26.6) ^a^	33 (51.6) ^b^	50 (39.1)	
Anti-thyroid	4 (6.3) ^a^	2 (3.1) ^a^	6 (4.7)	
Comorbidities				
None	57 (89.1)	56 (87.5)	113 (88.3)	0.531 ^f^
Hypertension	2 (3.1)	2 (3.1)	4 (3.1)	
Diabetes	3 (4.7)	6 (9.4)	9 (7)	
CVD	2 (3.1)	0 (0)	2 (1.6)	
Primary tumor side				
Right	29 (45.3)	33 (51.6)	62 (48.4)	0.469 ^f^
Left	32 (50)	25 (39.1)	57 (44.5)	
Isthmus	3 (4.7)	5 (7.8)	8 (6.3)	
Bilateral	0 (0)	1 (1.6)	1 (0.8)	
Primary tumor size				
<2 cm	40 (62.5)	36 (56.3)	76 (59.4)	0.404 ^f^
2–4 cm	22 (34.4)	22 (34.4)	44 (34.4)	
>4 cm	2 (3.1)	6 (9.4)	8 (6.3)	
Number of FNAs	2 (1–5)	2 (1–5)	2 (1–5)	0.747 ^m^
EU TI-RADS				
2	4 (6.3)	4 (6.3)	8 (6.3)	0.106 ^f^
3	31 (48.4)	19 (29.7)	50 (39.1)	
4	23 (35.9)	28 (43.8)	51 (39.8)	
5	6 (9.4)	13 (20.3)	19 (14.8)	
FT3	3 (1.9–4.26)	3 (2–3.82)	3 (1.9–4.26)	0.873 ^m^
FT4	1.11 (0.74–1.8)	1.1 (0.8–1.82)	1.1 (0.74–1.82)	0.855 ^m^
TSH	1.46 (0.01–6.7)	1.4 (0.01–5.6)	1.45 (0.01–6.7)	0.992 ^m^
Preoperative				
IG (%)	0.2 (0–1.3)	0.2 (0–1)	0.2 (0–1.3)	0.920
IG count (×109/L)	0.01 (0–0.09)	0.02 (0–0.1)	0.01 (0–0.1)	0.343
NLR	1.91 (0.86–6.45)	1.83 (1–15.13)	1.88 (0.86–15.13)	0.777
PLR	117.83 (56.84–278.74)	111.67 (71.99–329.17)	116.21 (56.84–329.17)	0.564
Postoperative				
IG (%)	0.2 (0–1.4)	0.2 (0–1)	0.2 (0–1.4)	0.521
IG count (×109/L)	0.01 (0–0.25)	0.01 (0–0.4)	0.01 (0–0.4)	0.188
NLR	1.82 (0.66–10.41)	1.79 (0.8–7.39)	1.8 (0.66–10.41)	0.276
PLR	126.65 (42.18–411.22)	116.22 (60.91–315.92)	120.33 (42.18–411.22)	0.990
∆IG (%)	0 (−0.9–0.4)	0 (−0.5–0.9)	0 (−0.9–0.9)	0.143
∆IG count (×109/L)	0 (−0.05–0.03)	−0.01 (−0.08–0.34)	0 (−0.08–0.34)	0.007

Abbreviations: CVD, cardiovascular disease; FNA, Fine-needle aspiration; FT3, free triiodothyronine; FT4, free thyroxine; IG: immature granulocytes; NLR, neutrophil-to-lymphocyte ratio; PLR, platelet-to-lymphocyte ratio; TI-RADS, thyroid imaging reporting and data system; TSH, thyroid stimulating hormone.

Bold values indicate statistically significant differences ( *p* < 0.05). ^t^ Independent two sample *t-* test, mean±SD; ^m^ ​​​​​Mann Whitney *U-* test, median (minimum - maximum); ^x^ Pearson’s chi-square test, ^f^ Fisher’s exact test, *n* (%); ^a and b^ No difference between groups with the same letter (Bonferroni-corrected *Z-* test).

No statistically significant difference in pre- or postoperative IG was found between the malignant and benign groups ( *p* >0.05 for both comparisons).

Delta values were calculated based on the difference between pre- and post-operative patient values. A significant difference in the delta IG counts between the groups was identified ( *p* =0.007). The median IG score was 0 in the benign patient group, compared with−0.01 in the malignant group ( Table 1 ).

**Fig. 1:**
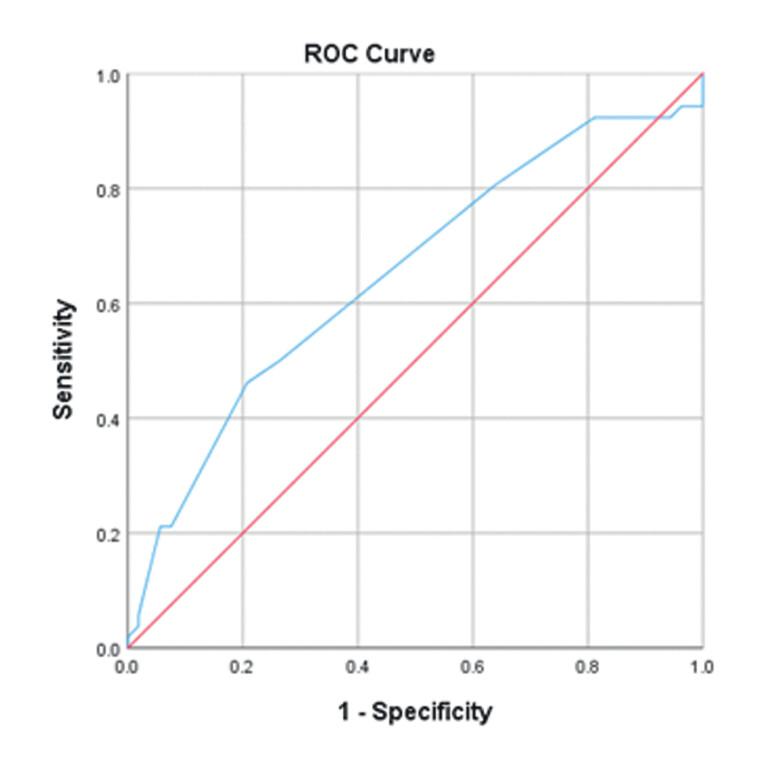
Receiver operating characteristic (ROC) curve of the delta IG count for predicting malignancy *Cut-off:≤− 0.01, sensitivity (%): 46.2, and specificity (%): 79.2. AUC, area under the curve; CI, confidence interval; IG, immature granulocytes; ROC, receiver operating characteristic.

The difference (delta) between pre- and postoperative IG counts emerging as associated with the patients’ malignancy status exhibited statistically adequate diagnostic accuracy ( *p* =0.008; Fig. 1 ).

At a delta IG count cut-off of≤− 0.01, the area under the curve (AUC; 95% confidence Interval [CI]) value was 0.651 (0.546–0.757), the sensitivity was 46.2%, and the specificity was 79.2% ( Fig. 1 ).

The effect level for the delta IG count in predicting malignancy was examined using univariate logistic regression models based on the cut-off obtained at ROC analysis in Fig. 1 . This was investigated first for the entire patient group and subsequently for those aged 55 years or above.

In the entire patient group, the probability of malignancy was higher in the group with a preoperative IG count difference of−0.01 or below than in those with a value greater than−0.01 (odds ratio [OR]=3.273, *p* =0.007). This shows that the risk of malignancy increases as the delta IG count decreases (becomes more negative). Similarly, this relationship was more marked in patients under 55 years of age (OR=5.082, *p* =0.007). However, the relationship was not significant in those aged 55 years or older ( *p* =0.372; Table 2 ).

**Table:** **Table 2** Univariate logistic regression analysis evaluating the association between delta IG count and malignancy (cut-off value: –0.01)

		SE	Wald	OR (95% CI)	*p*
Delta IG count (Ref.:>− 0.01)	All patients	1.186	0.438	3.273 (1.386–7.727)	**0.007**
	Age<55 y	0.599	7.376	5.082 (1.572–16.429)	**0.007**
	Age≥55 y	0.67	0.796	1.818 (0.489–6.763)	0.372

Abbreviations: CI, confidence interval; IG, Immature granulocyte; OR, odds ratio; SE, standard error. Bold values indicate statistically significant differences ( *p* < 0.05).

## Discussion

This study demonstrates that delta IG, representing the dynamic change in IG levels between pre- and postoperative measurements, was significantly associated with malignancy in AUS thyroid nodules. This finding supports our hypothesis that inflammatory markers reflecting bone marrow activation may serve as indicators of thyroid malignancy. 7​ Notably, while static preoperative or postoperative IG values showed no significant differences between groups, the temporal change emerged as a meaningful predictor, particularly in patients under 55 years of age.

A malignancy rate of 37.4% in our cohort aligns with the upper range of previously reported rates for AUS nodules. 9​ No significant relationship was detected with preoperative thyroid function tests, TI-RADS classification, or nodule size and laterality, although levothyroxine use was more common among malignant cases. These findings suggest that delta IG may provide complementary diagnostic information beyond conventional parameters, potentially serving as an accessible, cost-effective adjunct tool where molecular testing is unavailable, as highlighted in recent machine learning studies. 10​

Diagnostic uncertainty in thyroid malignancy is exemplified by rare cases of medullary thyroid carcinoma with negative calcitonin and carcinoembryonic antigen levels, 11​ underscoring the limitations of conventional biomarkers and the need for complementary, affordable inflammatory parameters such as delta IG. This uncertainty directly affects surgical decision-making as more radical procedures carry a higher risk of complications. 12​ Improving preoperative risk stratification with adjunctive markers may help avoid unnecessary aggressive surgery in low-risk patients while maintaining oncological safety.

Our findings extend prior investigations of inflammatory markers in thyroid malignancy. A recent study found no statistically significant hematological predictors in AUS patients. 10​ Similarly, our preoperative IG and other inflammatory markers showed no significant differences between groups. However, incorporating postoperative measurements revealed that dynamic changes, rather than single time-point values, may be more informative.

Additionally, the histological subtype distribution differed between studies. While follicular variant papillary thyroid carcinoma (FVPTC) comprised 48.3% of cancer cases in a previous study, 13​ classic variant papillary thyroid carcinoma was predominant in our cohort at 39.1%. This difference may contribute to variations in inflammatory marker profiles as FVPTC and classic PTC exhibit distinct biological behaviors and inflammatory responses. The heterogeneity of thyroid cancer subtypes within AUS nodules underscores the importance of developing biomarkers that perform across diverse histological presentations. 14​

Prior studies have included the age, tumor size, and particularly the K-TI-RADS classification as independent predictors of malignancy in patients under 55 years of age, although without postoperative evaluation. 5​ ​15​ In our study, the delta IG count emerged as a significant malignancy indicator in both the overall cohort and younger patients, suggesting utility across age groups. However, levothyroxine use was significantly more common in malignant cases (51.6%).

A retrospective study of 248 thyroid surgery patients found significant differences in preoperative neutrophil-to-lymphocyte ratio (NLR), delta neutrophil index, and IG counts between benign and malignant groups, but these differences disappeared postoperatively. 9​ Similarly, our study showed numerically higher preoperative IG counts in the malignant group, with equalization postoperatively, although this did not reach statistical significance. This may reflect our limited sample size and warrants evaluation in larger, multicenter studies.

When pre- and postoperative IG counts were compared as delta IG, a significant difference emerged in the malignant group, with the malignancy risk increasing as delta IG became more negative. This relationship was particularly pronounced in younger patients (<55 years) but not significant in older patients (≥55 years). The higher prevalence of comorbidities in older patients may have confounded the analysis. Furthermore, age-related changes, including increased chronic inflammation and decreased cell regeneration capacity, may diminish the sensitivity of inflammatory markers in elderly patients. 16​

A recent meta-analysis determined that although the NLR was more diagnostically useful than the platelet-to-lymphocyte ratio (PLR) for differentiating thyroid lesions, it exhibited only moderate diagnostic accuracy. Moreover, Gambardella et al. demonstrated that elevated NLR and PLR values were associated with an increased risk of malignancy in cytologically indeterminate nodules, suggesting that systemic inflammatory markers may contribute to preoperative risk stratification. However, their analysis relied exclusively on single preoperative measurements. In contrast, our findings indicate that dynamic changes in IG levels may provide additional discriminatory values beyond static indices. 16​ These markers were recommended as complementary tools alongside standard diagnostic methods. 17​

The broader clinical significance of inflammation in thyroid malignancy extends beyond diagnostic considerations. In aggressive histological subtypes, inflammatory burden has been associated with disease progression and survival outcomes. A multicenter retrospective analysis evaluating combined treatment strategies in anaplastic thyroid carcinoma demonstrated a close relationship between tumor biology, systemic condition, and clinical outcomes. 18​ These findings suggest that inflammation-related pathways may play a role not only in malignancy detection but also in tumor behavior and prognosis, further supporting the biological plausibility of dynamic inflammatory markers such as delta IG.

Similarly, the NLR and the PLR did not emerge as predictors of malignancy in our AUS cohort. Another study concluded that the NLR is a simple, reproducible inflammatory biomarker that can improve the preoperative malignancy risk assessment in indeterminate thyroid nodules. 16​ However, postoperative measurements were not performed. In our AUS cohort, the preoperative NLR showed no correlation with malignancy, and the postoperative NLR changes were similar in both groups, highlighting the need for larger studies incorporating both pre- and postoperative inflammatory marker assessments.

### Limitations

This study has several limitations. First, its single-center, retrospective design limits generalizability. Second, the relatively small sample size may have reduced statistical power to detect smaller effect sizes. Future prospective, multicenter studies with larger cohorts are needed to validate these findings.

Additionally, some patients had underlying thyroid disorders associated with chronic inflammation. These conditions may have influenced inflammatory markers and potentially affected the accuracy of malignancy prediction.

## Conclusions

Delta IG may serve as a clinically useful adjunct for preoperative risk stratification in AUS nodules, potentially helping to identify high-risk patients while reducing unnecessary interventions in low-risk cases. This approach could improve patient outcomes by minimizing overtreatment while optimizing resource utilization.

While these findings are promising, the limited sample size and single-center design necessitate cautious interpretation. Before clinical implementation, delta IG should be validated in larger, multicenter cohorts encompassing diverse patient populations to ensure broad applicability and clinical utility.
